# Combination of Alanine and Glutathione as Targeting Ligands of Nanoparticles Enhances Cargo Delivery into the Cells of the Neurovascular Unit

**DOI:** 10.3390/pharmaceutics12070635

**Published:** 2020-07-07

**Authors:** Gergő Porkoláb, Mária Mészáros, András Tóth, Anikó Szecskó, András Harazin, Zsolt Szegletes, Györgyi Ferenc, András Blastyák, Lajos Mátés, Gábor Rákhely, Mária A. Deli, Szilvia Veszelka

**Affiliations:** 1 Institute of Biophysics, Biological Research Centre, Temesvári krt. 62, H-6726 Szeged, Hungary; porkolab.gergo@brc.hu (G.P.); meszaros.maria@brc.hu (M.M.); toth.andras@brc.hu (A.T.); szecskoaniko@gmail.com (A.S.); harazin.andras@brc.hu (A.H.); szegletes.zsolt@brc.hu (Z.S.); rakhely.gabor@brc.hu (G.R.); 2 Department of Biotechnology, Faculty of Science and Informatics, University of Szeged, Közép fasor 52, H-6726 Szeged, Hungary; 3 Institute of Plant Biology, Biological Research Centre, Temesvári krt. 62, H-6726 Szeged, Hungary; ferenc.gyorgyi@brc.hu; 4 Institute of Genetics, Biological Research Centre, Temesvári krt. 62, H-6726 Szeged, Hungary; blastyak.andras@brc.hu (A.B.); mates.lajos@brc.hu (L.M.)

**Keywords:** blood–brain barrier, neurovascular unit, alanine, glutathione, nanoparticle, niosome, brain endothelial cell, pericyte, astrocyte, neuron

## Abstract

Inefficient drug delivery across the blood–brain barrier (BBB) and into target cells in the brain hinders the treatment of neurological diseases. One strategy to increase the brain penetration of drugs is to use vesicular nanoparticles functionalized with multiple ligands of BBB transporters as vehicles. Once within the brain, however, drugs must also be able to reach their therapeutic targets in the different cell types. It is, therefore, favorable if such nanocarriers are designed that can deliver their cargo not only to brain endothelial cells, but to other cell types as well. Here, we show that alanine-glutathione dual-targeting of niosomes enhances the delivery of a large protein cargo into cultured cells of the neurovascular unit, namely brain endothelial cells, pericytes, astrocytes and neurons. Furthermore, using metabolic and endocytic inhibitors, we show that the cellular uptake of niosomes is energy-dependent and is partially mediated by endocytosis. Finally, we demonstate the ability of our targeted nanovesicles to deliver their cargo into astroglial cells after crossing the BBB in vitro. These data indicate that dual-labeling of nanoparticles with alanine and glutathione can potentially be exploited to deliver drugs, even biopharmacons, across the BBB and into multiple cell types in the brain.

## 1. Introduction

Pharmaceutical treatment of neurological diseases is hindered by the inability of most drugs, especially biopharmacons, to cross the blood–brain barrier (BBB). To this day, numerous clinical trials have failed because of low penetration of therapeutics into the brain parenchyma [1]. One promising strategy to solve this problem is the use of nanoparticles (NPs) to deliver drugs into the central nervous system (CNS) [2,3]. Among nanocarriers, vesicular NPs are especially versatile as they are biocompatible, biodegradable, non-immunogenic and can accomodate both hydrophilic and lipophilic, high and low molecular weight cargoes, with controlled release properties [4,5]. Non-ionic surfactant-based vesicular NPs, so-called niosomes, have the additional advantage of being less sensitive to oxidation, therefore having greater physical stability than lipid-based vesicles [6].

Since NPs administered into the bloodstream get sequestered by several organs, especially the liver, spleen and kidneys, specific BBB-targeting of NPs is also needed to ensure sufficient brain delivery of therapeutics [7,8]. There is a large number of blood-to-brain transport systems at the cerebral endothelium that show a specific expression pattern [9,10,11] and can potentially be exploited to target NPs to the BBB. A major class of transporters that supply the CNS with nutrients are solute carriers (SLCs), a large family of proteins with many highly expressed members in brain endothelial cells [12,13,14]. Besides nutrients, SLCs transport clinically relevant small molecular drugs across the BBB [15] and are widely investigated as drug targets, yet these carriers and their ligands are not fully exploited for targeting NPs to the BBB [16,17]. Although its transporter(s) have not yet been identified, the antioxidant tripeptide glutathione is one of the most successful BBB targeting ligands of NPs. Glutathione PEGylated liposomes increased brain delivery of several small molecule drugs in different studies and are currently being tested in clinical trials [18,19,20]. In our previous study [21], we demonstrated that targeting niosomes with the SLC ligand alanine elevated the BBB penetration of a large protein cargo compared to non-targeted niosomes in vitro and in vivo. We also showed that dual ligand-labeling of niosomes with the combination of alanine and glutathione was even more effective to increase BBB transfer than single ligand-labeling with either alanine or glutathione [21].

However, penetration of NPs across the BBB still might not be enough for therapeutic success by itself. The endothelial layer of cerebral microvessels is covered with pericytes, ensheathed by astroglial endfeet and all these cells are in communication with neurons, collectively termed as the neurovascular unit (NVU) [22,23]. That means drugs have to travel through multiple cell layers to reach their therapeutic targets, usually in neurons or glial cells. It is, therefore, important that such nanocarriers are designed that can potentially deliver their cargo to each cell type of the NVU.

Despite this, little is known about the fate of targeted NPs in pericytes, astrocytes or neurons after having crossed the BBB. Gromnicova et al., for example, reported that glucose-coated, 4 nm gold NPs cross the brain endothelium and enter astrocytes [24], while other studies claim that polimeric or dendrimer-based nanocarriers are able to cross the BBB and may facilitate the delivery of small molecule drugs into astroglial cells or neurons [25,26]. To our best knowledge, however, there are no reports on targeted NPs successfully delivering large protein cargoes across the BBB and subsequently into pericytes, astrocytes or neurons. Furthermore, studies concerned with the uptake of a single type of targeted NP in multiple NVU cell types are scarce.

To fill this gap in our knowledge, our aim here was to test whether our previously described NPs targeting SLC transporters at the BBB [21] are also able to reach the cell types of the NVU. We show that alanine-glutathione dual-targeting of niosomes enhances the delivery of a large protein cargo (i) not only into brain endothelial cells but pericytes and astrocytes, as well as neurons, and (ii) into astroglial cells after crossing the BBB in vitro. Using metabolic and endocytic inhibitors, we also show that the process of cellular uptake is active and partially mediated by endocytosis. Our findings are further supported by gene expression data on SLCs, and characterization of niosomes as well as their effect on cell viability.

## 2. Materials and Methods

### 2.1. Animals

For primary cell isolations, brain tissues were obtained from 3-week-old and newborn outbred Wistar rats (Harlan Laboratories, Bicester, UK) of both sexes. The animals were fed on standard rodent chow and water ad libitum and were kept under a 12 h light/dark cycle in the conventional animal house of the Biological Research Centre, Szeged. Organ harvest from animals was performed following the regulations of the 1998. XXVIII. Hungarian law and the EU Directive 2010/63/EU about animal protection and welfare.

### 2.2. Materials and Reagents

All reagents were purchased from Sigma-Aldrich Kft. Hungary (part of Merck Life Science), unless otherwise indicated.

### 2.3. Cell Cultures

All cell cultures were grown in a humidified incubator at 37 °C with 5% CO_2_. For morphological characterization, phase-contrast microscopy images were taken of cultures of each cell type with a Motic AE2000 inverted microscope (Motic Instruments, Wetzlar, Germany).

#### 2.3.1. Primary Cell Cultures

Isolation of primary rat brain endothelial cells, pericytes and astrocytes were performed according to the method described in our previous studies [27,28]. After isolation, brain endothelial cells were seeded onto culture dishes (Corning Costar, New York, NY, USA) coated with collagen type IV (100 μg/mL) and fibronectin (25 μg/mL) and were cultured in DMEM/HAM’s F-12 (Gibco, Waltham, MA, USA) supplemented with 15% plasma-derived bovine serum (PDS, First Link, UK), 10 mM HEPES, 100 μg/mL heparin, 5 μg/mL insulin, 5 μg/mL transferrin, 5 ng/mL sodium selenite (ITS, Pan-Biotech, Aidenbach, Germany), 1 ng/mL basic fibroblast growth factor (bFGF, Roche, Basel, Switzerland) and 50 μg/mL gentamicin. During the first 3 days of culture, the medium of endothelial cells also contained 3 μg/mL puromycin to eliminate P-glycoprotein negative, contaminating cell types [29]. After the first 3 days of culture, the amount of PDS was decreased from 15% to 10% in the culture medium.

**Figure 1:**
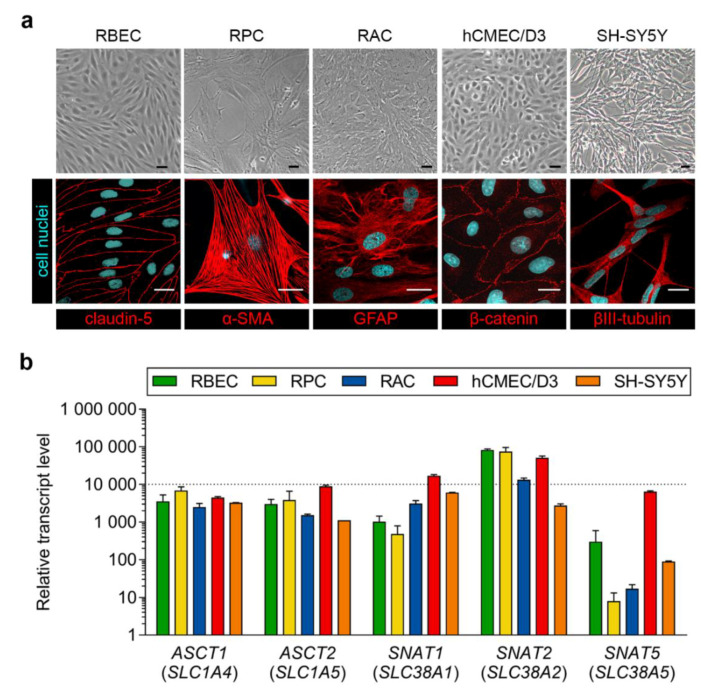
(**a**) Phase-contrast microscopy images and immunostainings of cultured primary rat brain endothelial cells (RBEC), primary rat pericytes (RPC), primary rat astrocytes (RAC), hCMEC/D3 human brain endothelial cells and SH-SY5Y human neurons. α-SMA: α-smooth muscle actin; GFAP: glial fibrillary acidic protein. Bar: 25 µm. (**b**) Expression of genes encoding alanine transporters in the same cell types. RBEC and hCMEC/D3 data is shown with permission from [15]. Values presented are means ± SEM.

After isolation, pericytes were seeded onto culture dishes (VWR International, Radnor, PA, USA) coated with collagen type IV (100 μg/mL), whereas astrocytes were plated onto uncoated 75 cm^2^ flasks (TPP, Trasadingen, Switzerland). Both pericytes and astrocytes were cultured in DMEM medium (low glucose, Gibco, Waltham, MA, USA) supplemented with 10% fetal bovine serum (FBS, Pan-Biotech, Aidenbach, Germany) and 50 μg/mL gentamicin. Glial cells gave positive immunostaining for glial fibrillary acidic protein (GFAP) and pericytes for α-smooth muscle actin (α-SMA) as shown in Figure 1b, in accordance with our previous studies [27,30] and literature data [31].

#### 2.3.2. Cell Lines

The hCMEC/D3 human brain endothelial cell line was purchased from Merck Millipore. Cultures of hCMEC/D3 (passage number ≤ 35) were grown in MCDB 131 medium (Pan-Biotech, Aidenbach, Germany) supplemented with 5% FBS (Sigma, Darmstadt, Germany), GlutaMAX (100×, Life Technologies, Carlsbad, CA, USA), lipid supplement (100×, Life Technologies, Carlsbad, CA, USA), 10 mM HEPES, 5 μg/mL insulin, 5 μg/mL transferrin, 5 ng/mL sodium selenite (ITS, Pan-Biotech, Aidenbach, Germany), 100 μg/mL heparin, 10 μg/mL ascorbic acid, 550 nM hydrocortisone, 1 ng/mL bFGF (Roche, Basel, Switzerland) and 50 μg/mL gentamicin [32]. Before each experiment the culture medium of hCMEC/D3 cells were supplemented with 10 mM LiCl for 24 h to improve barrier properties [33].

The SH-SY5Y human neuroblastoma cell line was purchased from ATCC (ATCC CRL-2266). Cultures of SH-SY5Y were grown in DMEM/HAM’s F-12 medium (Gibco, Waltham, MA, USA) supplemented with 10% FBS (Pan-Biotech, Aidenbach, Germany) until reaching confluency. As undifferentiated SH-SY5Y cells can grow as epithelioid cells or neuroblasts, we used a retinoic acid-based differentation method [34]. Confluent cultures were differentiated for 5 days in a medium containing DMEM/HAM’s F-12 (Gibco, Waltham, MA, USA) supplemented with 2% FBS (Pan-Biotech, Aidenbach, Germany), 0.5% dimethyl-sulfoxide (DMSO) and 10 µM retinoic acid to induce a more branched, neuronal phenotype in SH-SY5Y cells [34]. For experiments, only differentiated cultures of SH-SY5Y were used.

### 2.4. Immunohistochemistry

For immunohistochemical characterization, all cell types were cultured on rat tail collagen-coated glass cover slips (borosilicate; VWR International, Radnor, PA, USA). Primary rat brain endothelial cells were stained for claudin-5, pericytes for α-SMA and astroglial cells for GFAP. hCMEC/D3 cells were stained for β-catenin and SH-SY5Y cells for βIII-tubulin. After removing the culture medium, cells were fixed with ice cold methanol-acetone 1:1 solution at −20 °C for 5 min (claudin-5, β-catenin) or with 4% paraformaldehyde at room temperature for 10 min (α-SMA, GFAP, βIII-tubulin), blocked with 3% bovine serum albumin (BSA) diluted in phosphate buffer (PBS) for 1 h at room temperature and incubated with primary antibodies at 4 °C overnight. Primary antibodies used were rabbit anti-claudin-5 (1:200; Sigma), mouse anti-α-SMA (1:300, Agilent Dako, Santa Clara, CA, USA), goat anti-GFAP (1:1000; Abcam, Cambridge, UK), rabbit anti-β-catenin (1:200, Agilent Dako, Santa Clara, CA, USA) and mouse anti-β-tubulin (1:250; Invitrogen, Life Technologies, Carlsbad, CA, USA). Cells were then incubated with secondary antibodies (Cy3-labeled anti-rabbit and Alexa Fluor 488-labeled anti-mouse and anti-goat, Invitrogen, Life Technologies, Carlsbad, CA, USA) as well as Hoechst 33342 (1 µg/mL) for nuclei staining for 10 min at room temperature. Between each incubation, cells were washed 3 times with PBS. After mounting the samples (Fluoromount-G, Southern Biotech, Birmingham, AL, USA), stainings were examined with a Leica TCS SP5 confocal laser scanning microscope (Leica Microsystems, Wetzlar, Germany).

### 2.5. RNA Isolation and Quality Control

In the case of primary rat brain endothelial cells, pericytes, astrocytes and hCMEC/D3 cells, total RNA isolation and quality control was performed according to the method described in our previous studies [15,35]. Shortly, cells were cultured for 5 days, after reaching confluency washed with ice cold PBS, scraped and collected. Total RNA was isolated from cell pellets using RNAqueous-4PCR Kit (Ambion, Life Technologies, Carlsbad, CA, USA) with DNase1 (RNase-free) treatment according to the manufacturer’s instructions. In the case of SH-SY5Y cells, total RNA was isolated from cell pellets using TRI reagent, and the concentration and purity of RNA samples were assessed by a NanoDrop ND-1000 spectrophotometer (NanoDrop Technologies, Wilmington, DE, USA).

### 2.6. Quantitative Real-Time Polymerase Chain Reaction and Data Analysis

For all samples, cDNA synthesis was performed on 1 μg total RNA samples by High Capacity cDNA Reverse Transcription Kit (Life Technologies, Carlsbad, CA, USA) using random hexanucleotide primers and MultiScribe Reverse Transcriptase in the presence of RNase inhibitor according to the manufacturer’s instructions. The expression of genes encoding alanine transporters (sodium-coupled neutral amino acid transporters *SNAT1/SLC38A1*, *SNAT2/SLC38A2* and *SNAT5/SLC38A5*; neutral amino acid transporters *ASCT1/SLC1A4* and *ASCT2/SLC1A5*) were analyzed by quantitative real-time polimerase chain reaction (qPCR) using TaqMan Low Density Array 384-well microfluidic cards preloaded with TaqMan Gene Expression Assays (Life Technologies, Carlsbad, CA, USA) [15]. qPCRs were performed using ABI TaqMan Universal Master Mix (Applied Biosystems, Life Technologies, Carlsbad, CA, USA) by the ABI Prism 7900 system (Applied Biosystems, Life Technologies, Carlsbad, CA, USA). qPCR data were analyzed using the ABI SDS 2.0 software (Applied Biosystems, Life Technologies, Carlsbad, CA, USA).

For SH-SY5Y cells, the expression of the above mentioned genes were analyzed by qPCR using TaqMan Gene Expression Assays (Hs01562175_m1, Hs01089954_m1, Hs01012028_m1, Hs00161719_m1, Hs00194540_m1, respectively; Applied Biosystems, Life Technologies, Carlsbad, CA, USA). For this cell type, qPCR was performed using TaqMan Gene Expression Master Mix (Applied Biosystems, Thermo Fisher Scientific, Waltham, MA, USA) by the ABI Prism 7500 system (Applied Biosystems, Life Technologies, Carlsbad, CA, USA). qPCR data were analyzed using the ABI SDS 1.4 software (Applied Biosystems, Life Technologies, Carlsbad, CA, USA).

In all cases, the expression of genes was normalized to 18S rRNA, which was used as an endogenous control (ΔC_t_ = C_tgene_ − C_t18S rRNA_). In all samples, rRNA was quantified by qPCR using TaqMan Gene Expression Assay (Hs99999901_s1) (Applied Biosystems, Thermo Fisher Scientific, Waltham, MA, USA). Expression values of studied genes were determined based on the normalized expression of genes calculated with 2^−ΔCt^ formula, and were correlated to the lowest normalized expression measured by the applied qPCR method, as in our previous studies [35,36].

### 2.7. Synthesis of the Targeting Ligands of Niosomes

Dodecanoyl alanine was prepared according to Liu et al. [37]. Briefly, 100 mL NaOH (1 M) and 1.34 g (0.015 mol) L-alanine were added into a one-neck flask. The system was cooled to 0 °C, then 3.31 mL (0.014 mol) dodecanoyl chloride was added dropwise to the mixture and maintained at 0 °C for 5 h. After that, 16 mL hydrochloric acid (12 M) was added to the reaction and the white precipitate was filtrated. Finally, the product was washed three times with deionized water and dried at 45 °C for 24 h.

For the synthesis of DSPE-PEG-glutathione, 13.5 mg glutathione (0.044 mM) was reacted with 100 mg DSPE-PEG-maleimide (0.035 mM) (N-[(3-Maleimide-1-oxopropyl) aminopropyl polyethyleneglycol-carbamyl] distearoylphosphatidyl-ethanolamine, SUNBRIGHT^®^ DSPE-020MA [DSPE-PEG-MAL], purchased from NOF Europe, Belgium) in 0.1 M ammonium acetate for a day under nitrogen. The product was lyophilized three times to remove ammonium acetate [21].

### 2.8. Preparation of Niosomes

Niosomes were prepared as described in our previous paper [21], with minor modifications. For the preparation of non-targeted niosomes (N), non-ionic surfactants Span 60 (sorbitane-monostearate) and Solulan C24 (cholesteryl-poly-24-oyxyethylene-ether, Chemron Co., Avon, OH, USA), as well as cholesterol were dissolved in a hot mixture of chloroform and ethanol (1:2) in a round-bottom flask. To prepare alanine-glutathione dual-targeted niosomes (N-A-GSH), dodecanoyl-alanine (A, 4 *w/w*% of total lipid) and DSPE-PEG-GSH (GSH, 4 w/w% of total lipid) were also added to the mixture before the dissolving step. The removal of organic solvents by a vacuum pump yielded a thin lipid film layer. For cellular uptake studies, the dry lipid film was hydrated with phosphate buffer (PBS; KCl 2.7 mM, KH_2_PO_4_ 1.5 mM, NaCl 136 mM, Na_2_HPO_4_ × 2 H_2_O 6.5 mM, pH 7.4) containing Evans blue-labeled bovine serum albumin (EBA, 67 kDa; 0.167 mg/mL EB, 10 mg/mL BSA) as in our previous study [21]. For visualization of cellular uptake as well as permeability studies, PBS containing mCherry (26.7 kDa; 5 mg/mL), a red fluorescent protein with better properties for confocal microscopy than EBA, was used to hydrate the lipid film. For purification of recombinant mCherry protein, cDNA was subcloned into pPROEX HTb, and 6HIS-tagged mCherry was expressed in BL21(DE3)pLysS. Purification was performed on Ni-NTA matrix according to standard procedures with subsequent and extensive dialysis against PBS to remove imidazole. 

After this, the hydration step mixtures were sonicated in a water bath for 60 min at 45 °C. To obtain vesicles the suspension was forced through a polycarbonate filter (Whatman filter, 13 mm, 100 nm pore size) by lipid extrusion technique (high pressure thermobarrel extruder, Lipex Biomembranes Inc., Vancouver, BC, Canada). The non-encapsulated cargo was removed by ultracentrifugation (123,249× *g*, 4 °C, 6 h for EBA or 3 h for mCherry), the pelleted niosomes were resuspended in PBS for size and zeta potential measurements or phenol red-free DMEM/HAM’s F-12 medium for experiments and stored at 4 °C.

### 2.9. Characterization of Niosomes

#### 2.9.1. Atomic Force Microscopy

Atomic force microscopy measurements were carried out with an Asylum Research MFP-3D head and controller (Oxford Instruments, Asylum Research, UK). The driver program of MFP-3D (version 16.12.214) was written in IGOR Pro Software (version 6.38B01, Wavemetrics). Image procession and data calculation were made using the same software. For imaging, gold-coated silicon nitride rectangular cantilevers were used with a typical spring constant of 0.03 N/m (Bruker OBL 10). The spring constant for each cantilever was determined by thermal fluctuation technique [38], followed by Sader’s method [39]. For measurements, freshly cleaved mica (SPI-Chem Mica Sheets) surfaces were incubated in 2% APTES ((3-aminopropyl) triethoxysilan, Sigma) dissolved in isopropanol to create free amine groups on their surface [40]. Niosomes were attached to the modified surface with glutaraldehyde. Measurements were carried out in tapping (AC) mode in phosphate buffer. Typically, 512 × 512 point scans were taken at 0.5 Hz scan rate. Both the trace and retrace images were recorded and compared. The measurements presented here are 10 × 10 µm^2^ flattened heights.

#### 2.9.2. Size, Size Stability and Zeta Potential Measurements

Particle size, polydispersity index (PDI) and zeta potential of niosomes were measured by dynamic light scattering (Malvern Zetasizer Nano ZS, Malvern, UK). Before measurements, samples were diluted in PBS to a final concentration of 2 mg/mL. Means were calculated from the average of at least 3 × 13 measurements per sample. To determine the size stability of niosomes over time, particle size and PDI were monitored every week for 6 weeks at 2 mg/mL concentration. To assess the size stability of niosomes at increasing concentrations, samples were diluted to either 2, 4, 6, 8 or 10 mg/mL in PBS. To determine the size stability of niosomes in serum, samples were diluted to either 2 or 10 mg/mL concentrations in PBS containing 10% FBS. Because multiple peaks were detected in serum samples (without NPs) and a multimodal distribution was also seen in samples of NPs containing serum (Supplementary Figure S1), we used the mean diameter of the largest peak to assess the serum stability of NPs.

### 2.10. Cell Viability Assay

Kinetics of cellular responses to niosome treatments were monitored by impedance measurement (RTCA-SP; ACEA Biosciences, San Diego, CA, USA), which is label-free, real-time, non-invasive test and correlates linearly with viability of cells [41]. Furthermore, this method correlates well with other conventional cell viability assays, as shown in our previous article [42]. After background measurements with culture medium, cells were seeded at a density of 6 × 10^3^ cells/well in 96-well plates with integrated gold electrodes (E-plate 96, ACEA Biosciences, San Diego, CA, USA). The wells were coated with collagen type IV (100 µg/mL) and, in the case of primary rat brain endothelial cells, with collagen type IV (100 µg/mL) and fibronectin (25 µg/mL). When the growth of cultures reached a plateau phase, cells were treated with niosomes (1, 3 or 10 mg/mL) and their impedance was monitored at every 5 min for either 4 h or 24 h (depending on the length of further experiments). Triton X-100 detergent (1 mg/mL) was used as a reference compound inducing maximum cell toxicity. Cell index was defined as R_n_ − R_b_ at each time point of measurement, where R_n_ is the impedance of the well when it contains adherent cells and R_b_ is the background impedance of the well containing culture medium alone. Cell index was normalised in each well to the value measured at the last timepoint before the treatment.

### 2.11. Cellular Uptake Studies

For quantification of cellular uptake, all cell types were cultured in 24-well plates (Corning Costar, New York, NY, USA). Cultures were incubated with 10 mg/mL niosome solutions (N or N-A-GSH containing EBA cargo) diluted in the respective culture medium of each cell type at 37 °C for 4 h (according to [19,21]) on a horizontal shaker (150 rpm). To elucidate uptake mechanisms, cells were either incubated with niosomes at 4 °C for 4 h, co-incubated with niosomes and metabolic inhibitor sodium azide (1 mg/mL) at 37 °C for 4 h or pre-treated with endocytosis inhibitors filipin (5 μg/mL, 15 min) or cytochalasin D (0.125 μg/mL, 1 h) and then incubated with niosomes at 37 °C for 4 h. After incubation with niosomes, cells were washed three times with ice cold PBS supplemented with 0.1% BSA, once with acid stripping buffer (glycine 50 mM, NaCl 100 mM, pH 3) to remove cell surface-associated niosomes and once with PBS. Finally, cells were lysed in distilled water containing 10 mg/mL Triton X-100 detergent and the fluorescent signal of EBA cargo was quantified with a spectrofluorometer (Horiba Jobin Yvon Fluorolog 3, USA) at 584 nm excitation and 663 nm emission wavelengths [21].

For visualization of cellular uptake, all cell types were cultured in glass bottom culture dishes (diameter: 3.5 cm, Greiner Bio-One, Germany). Confluent monolayers were incubated with either mCherry in solution (2 μg/mL) or niosomes containing mCherry cargo (10 mg/mL N or N-A-GSH, containing 2 μg/mL mCherry) diluted in the respective culture medium of each cell type at 37 °C for 4 h. For the staining of cell nuclei, Hoechst 33,342 dye (1 μg/mL, 10 min) was used. After incubation the culture medium was removed and cells were washed with Ringer-HEPES buffer (118 mM NaCl, 4.8 mM KCl, 2.5 mM CaCl_2_, 1.2 mM MgSO_4_, 5.5 mM d-glucose, 20 mM HEPES, pH 7.4) supplemented with 1% PDS. Visualization of mCherry cargo in living cells was done with a Leica TCS SP5 confocal laser scanning microscope (Leica Microsystems, Wetzlar, Germany).

### 2.12. Permeability Studies

For permeability studies we used a well characterized triple co-culture blood-brain barrier (BBB) model in which primary rat brain endothelial cells, pericytes and astrocytes are cultured together in a transwell system [15,27]. Astroglial cells were passaged (8.5 × 10^4^ cells/cm^2^) to glass bottom 12-well plates (the plastic bottom of standard 12-well plates from Corning Costar, New York, NY, USA was replaced by borosilicate glass coverslips from VWR International, Radnor, PA, USA) coated with Matrigel (growth factor reduced, Corning Costar, New York, NY, USA). To prepare the co-culture model, pericytes at P2 were passaged (1.5 × 10^4^ cells/cm^2^) to the bottom side of tissue culture inserts (Transwell, polycarbonate membrane, 3 μm pore size, Corning Costar, New York, NY, USA) coated with collagen type IV (100 μg/mL). Brain endothelial cells were seeded (7.5 × 10^4^ cells/cm^2^) to the other, upper side of the culture insert membrane coated with Matrigel (growth factor reduced). Then the inserts containing brain endothelial cells and pericytes on the two sides of the membrane were placed in 12-well plates containing astrocytes at the bottom. Both the upper and lower fluid compartments of the model received endothelial cell culture medium supplemented with 550 nM hydrocortisone. The three cell types were cultured together for 4 days before permeability experiments began.

The tightness of the BBB model was verified by measurements of transendothelial electrical resistance (TEER) by an EVOM voltohmmeter (World Precision Instruments, Sarasota, FL, USA) combined with STX-2 electrodes. When TEER values of ≥250 Ω × cm^2^ were obtained, the model was used for experiments. The upper, donor compartment (0.5 mL) was incubated with either 2 μg/mL mCherry solution or niosomes containing mCherry cargo (10 mg/mL N or N-A-GSH, containing 2 μg/mL mCherry) diluted in phenol red-free DMEM/HAM’s F-12 medium (Gibco, Waltham, MA, USA) supplemented with 1% PDS at 37 °C for 24 h on a horizontal shaker (150 rpm). To assess the integrity of the model, the paracellular marker sodium fluorescein (SF; 376 Da, 10 μg/mL) and the transcellular marker EBA (67 kDa, 10 mg/mL BSA + 167.5 μg/mL Evans blue) were also tested for permeability. After incubation, samples were collected from both compartments and the fluorescent signal of mCherry cargo was quantified at 582 nm excitation and 605 nm emission wavelengths with a spectrofluorometer (Horiba Jobin Yvon Fluorolog 3, Piscataway, NJ, USA). The fluorescent signal of the marker molecules were quantified at 485 nm excitation and 520 nm emission wavelengths (SF) and 584 nm excitation and 680 nm emission wavelengths (EBA) by a fluorescence multiwell plate reader (Fluostar Optima, BMG Labtechnologies, Ortenberg, Germany). The apparent permeability coefficients (P_app_) were calculated as described previously [43] with the following equation:

$$\frac{\Delta {\left[C\right]}_{A}{\;\times \;V}_{A}}{\;A\;\times \;{\left[C\right]}_{D}\;\times \;\Delta t}$$

Briefly, P_app_ (cm/s) was calculated from the concentration difference of the cargo in the acceptor compartment (Δ[C]_A_) after 24 h. [C]_D_ is the concentration in the donor compartment at 0 h, V_A_ is the volume of the acceptor compartment (1.5 mL), and A is the surface area available for permeability (1.12 cm^2^).

For the visualization of mCherry that crossed the BBB model during the 24 h permeability assay and was subsequently taken up by astrocytes, living cells at the bottom of the 12-well plates were washed with Ringer-HEPES buffer supplemented with 1% PDS and immediately examined with a Leica TCS SP5 confocal laser scanning microscope. For the staining of cell nuclei, Hoechst 33342 dye (1 μg/mL, 10 min) was used.

### 2.13. Statistical Analysis

Data are presented as means ± SEM or SD. Statistical analyses were performed using GraphPad Prism 8 software (GraphPad Software, San Diego, CA, USA). Means were compared using unpaired t test or one-way ANOVA followed by Dunnett’s posttest. Differences were considered statistically significant at *p* < 0.05. All experiments were repeated at least two times and the number of parallel samples in each experiment was 3–8.

## 3. Results

### 3.1. Expression of Genes Encoding Alanine Transporters in the Cell Types of the Neurovascular Unit

As a general characterization of the cell types used in this study, Figure 1a shows phase-contrast microscopy and immunohistochemical staining images of primary rat brain endothelial cells, pericytes and astrocytes, as well as hCMEC/D3 human brain endothelial cells and differentiated SH-SY5Y human neurons. In these cultures we verified the expression of five genes encoding solute carrier (SLC) transporters that carry the amino acid alanine into cells (Figure 1b). Among alanine transporter genes, small neutral amino acid transporter *SNAT2* (*SLC38A2*) was highly expressed in all cell types but SH-SY5Y neurons. Neutral amino acid transporter genes *ASCT1* (*SLC1A4*) and *ASCT2* (*SLC1A5*) as well as *SNAT1* (*SLC38A1*) were expressed at moderate levels, whereas the expression of *SNAT5* (*SLC38A5*) was low-to-moderate in all cell types tested.

### 3.2. Characterization of Niosomes

**Figure 2:**
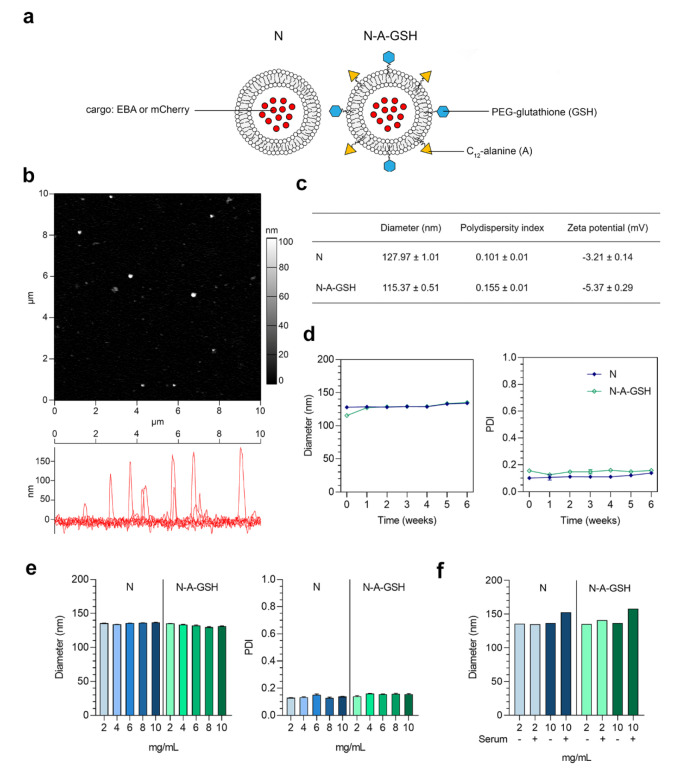
(**a**) Schematic drawing of non-targeted (N) and alanine-glutathione targeted (N-A-GSH) niosomes. EBA: Evans blue-albumin complex. (**b**) Atomic force microscopy image of N-A-GSH niosomes. (**c**) Main physico-chemical properties of niosomes. Values presented are means ± SD. (**d**) Size stability of niosomes over time at 2 mg/mL concentration, (**e**) at week 12 with increasing NP concentrations and (**f**) in PBS with or without 10% FBS. PDI: polydispersity index. Values presented are means ± SD.

A schematic drawing of non-targeted (N) and alanine-glutathione dual-targeted (N-A-GSH) niosomes is presented in Figure 2a. The morphology of niosomes appeared to be spherical as observed by atomic force microscopy and no aggregation of the particles at 2 mg/mL concentration was visible (Figure 2b). The mean diameter of niosomes was around 128 and 115 nm for N and N-A-GSH groups, respectively, and we measured a relatively narrow size distribution by dynamic light scattering, indicated by polydispersity index (PDI) values below 0.16 in both groups (Figure 2c). The zeta potential of non-targeted niosomes was slightly negative, whereas N-A-GSH niosomes had a more negative surface charge (Figure 2c), which is in concordance with our previous observations [21]. 

We also assessed the size stability of our NPs over time for 6 weeks (Figure 2d), and at one time point with increasing concentrations (Figure 2e). In these experiments, the mean diameter and PDI of niosomes changed moderately and reached a maximum diameter of approximately 135 nm and a PDI of 0.15. Both non-targeted and targeted NPs retained their size well when measured in PBS containing 10% FBS (Figure 2f), with a 15–20 nm increase in diameter at 10 mg/mL, indicating good stability in serum as well.

### 3.3. Effect of Niosomes on Cell Viability

**Figure 3:**
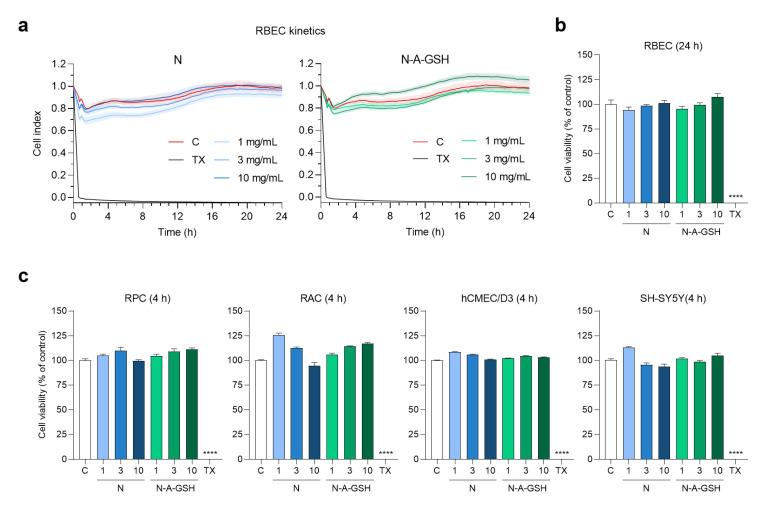
Effect of non-targeted (N) and alanine-glutathione dual-targeted (N-A-GSH) niosomes at concentrations of 1, 3 or 10 mg/mL on the viability of NVU cells monitored by real-time impedance measurement. (**a**) Kinetics of rat brain endothelial cell responses to niosome treatments for 24 h. Values presented are means ± SEM and are given as cell index. (**b**) Rat brain endothelial cell viability after 24 h. (**c**) Cell viability of pericytes (RPC), astrocytes (RAC), hCMEC/D3 endothelial cells and SH-SY5Y neurons after 4 h. Values presented are means ± SEM and are given as a percentage of control. Statistical analysis: one-way ANOVA followed by Dunnett’s posttest; **** *p* < 0.0001 compared to the control group; *n* = 6–8. C: culture medium-treated control group; TX: Triton X-100 reagent, indicating maximum cellular toxicity.

To determine a safe treatment concentration of NPs for further experiments, we monitored cellular responses to incubation with 1, 3 or 10 mg/mL niosomes by real-time impedance measurements. Kinetics of rat brain endothelial cell responses to NP treatments are shown in Figure 3a. At 24 h (the length of permeability experiments), none of N or N-A-GSH treatments decreased the impedance of brain endothelial cell layers compared to the culture medium-treated control group (Figure 3b). This indicates good cell viability and is in agreement with our previous results [21]. At 4 h (the length of uptake experiments), none of the NP treatments decreased the impedance of pericyte, astrocyte, hCMEC/D3 endothelial cell or SH-SY5Y neuronal cell layers (Figure 3c). For further treatments, therefore, we selected the 10 mg/mL concentration of niosomes, which could be considered as safe for both N and N-A-GSH groups.

### 3.4. Cellular Uptake of Cargo: Pericytes

**Figure 4:**
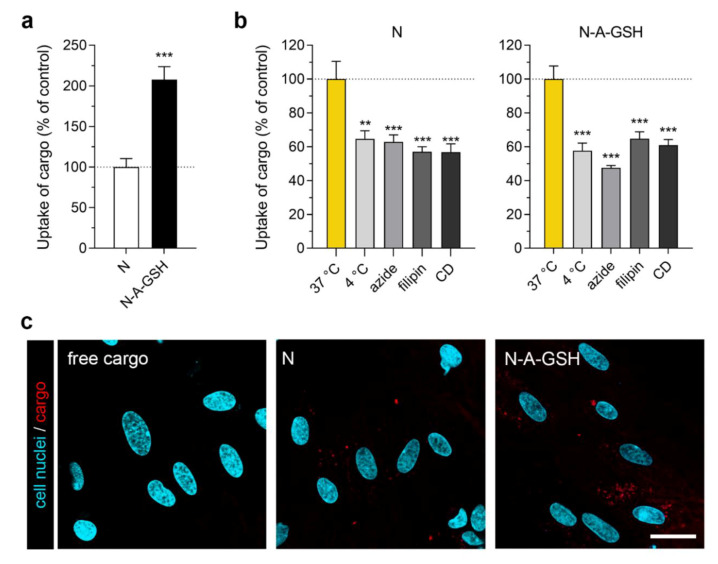
Cellular uptake of niosome cargo in cultured primary rat pericytes (RPC) after 4 h of incubation. (**a**) Uptake of cargo loaded in non-targeted (N) and alanine-glutathione targeted (N-A-GSH) niosomes. Values presented are means ± SEM. Statistical analysis: unpaired t test; *** *p* < 0.001; *n* = 4–6. (**b**) Effect of temperature and treatment with sodium-azide (1 mg/mL), filipin (5 µg/mL) or cytochalasin D (CD; 0.125 µg/mL) on the cellular uptake of cargo. Values presented are means ± SEM. Statistical analysis: one-way ANOVA followed by Dunnett’s posttest; ** *p* < 0.01, *** *p* < 0.001 compared to the first column of each niosome group; *n* = 6. (**c**) Live cell visualization of cargo taken up by pericytes. Free cargo: cargo not loaded in niosomes. Bar: 25 µm.

In primary rat pericytes, the uptake of EBA cargo encapsulated in dual-targeted niosomes was more than twice as high (208%) as cargo encapsulated in non-targeted niosomes after 4 h of incubation (Figure 4a). The amount of EBA cargo taken up by cells normalized to cargo inside niosome treatment solutions (mg/mg) is provided for both NP groups in each cell type in Supplementary Table S1. To test the temperature- and energy-dependency of the uptake process, we performed the experiment at 4 °C or co-treated the cells with niosomes and sodium azide (1 mg/mL) at 37 °C (Figure 4b). At 4 °C, active uptake processes are blocked in cells, whereas sodium azide is an inhibitor of adenosine triphosphate (ATP) synthesis [44]. These treatments significantly decreased the uptake of cargo in brain pericytes in both NP groups (N_4 °C_: 65%, N_azide_: 63%; N-A-GSH_4 °C_: 58%, N-A-GSH_azide_: 48%), suggesting an active cellular process. To further elucidate the mechanism of cellular uptake, we pre-treated the cells with inhibitors of endocytosis, filipin (5 µg/mL, 15 min) or cytochalasin D (CD; 0.125 µg/mL, 1 h). Filipin is an inhibitor of lipid raft/caveolae-mediated endocytosis, whereas cytochalasin D is an actin polymerization-blocking agent inhibiting all major endocytic routes [45]. When endocytic processes were blocked in brain pericytes, the uptake of EBA was lower than in the control group (N_filipin_: 57%, N_CD_: 57%; N-A-GSH_filipin_: 65%, N-A-GSH_CD_: 61%), suggesting a role of endocytosis in the uptake of NP cargo (Figure 4b).

We also visualized mCherry cargo taken up by living pericytes after 4 h of incubation (Figure 4c). In agreement with our spectrophotometry data in Figure 4a, a higher amount of cargo (indicated by red dots) could be observed inside cells treated with N-A-GSH niosomes compared to N niosomes. Red fluorescence was barely detectable in cells treated with non-encapsulated (free) cargo. To confirm that mCherry cargo is indeed present inside, rather than between cells, we provide the fluorescent, brightfield and merged channels of all confocal microscopy images used in this study in Supplementary Figure S2.

### 3.5. Cellular Uptake of Cargo: Astrocytes

**Figure 5:**
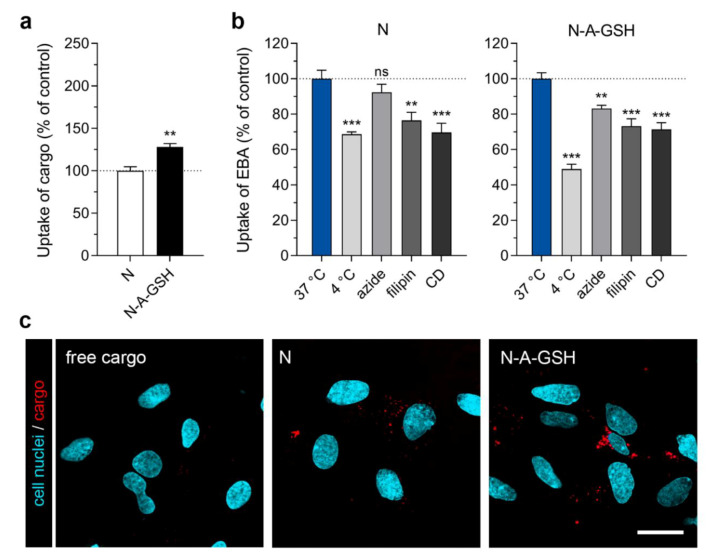
Cellular uptake of niosome cargo in cultured primary rat astrocytes (RAC) after 4 h of incubation. (**a**) Uptake of cargo loaded in non-targeted (N) and alanine-glutathione targeted (N-A-GSH) niosomes. Values presented are means ± SEM. Statistical analysis: unpaired t test; ** *p* < 0.01; *n* = 6. (**b**) Effect of temperature and treatment with sodium-azide (1 mg/mL), filipin (5 µg/mL) or cytochalasin D (CD; 0.125 µg/mL) on the cellular uptake of cargo. Values presented are means ± SEM. Statistical analysis: one-way ANOVA followed by Dunnett’s posttest; ** *p* < 0.01, *** *p* < 0.001, ns: not significant (*p* > 0.05) compared to the first column of each niosome group; *n* = 6. (**c**) Live cell visualization of cargo taken up by astrocytes. Free cargo: cargo not loaded in niosomes. Bar: 25 µm.

The uptake of cargo encapsulated in N-A-GSH niosomes was also higher in primary rat astrocytes (128% compared to cargo encapsulated in non-targeted niosomes; Figure 5a). Performing the experiment at 4 °C or co-treating the cells with sodium-azide decreased the uptake of cargo in both groups (N_4°C_: 69%, N_azide_: 92%, *p* > 0.05; N-A-GSH_4°C_: 49%, N-A-GSH_azide_: 83%; Figure 5b). Treatment of astroglial cells with endocytic inhibitors filipin and cytochalasin D (CD) also reduced cargo uptake (N_filipin_: 76%, N_CD_: 70%; N-A-GSH_filipin_: 73%, N-A-GSH_CD_: 71%; Figure 5b). These data together suggest an energy-dependent uptake mechanism that is partially mediated by endocytosis.

Figure 5c shows the live cell visualization of mCherry cargo taken up by astrocytes after 4 h of incubation. Only a small amount of mCherry could be observed in cells treated with non-encapsulated cargo. More red fluorescence could be seen in cells treated with non-targeted niosomes, whereas alanine-glutathione dual-targeted niosomes delivered the highest amount of cargo into astroglial cells.

### 3.6. Cellular Uptake of Cargo: hCMEC/D3 Brain Endothelial Cells

**Figure 6:**
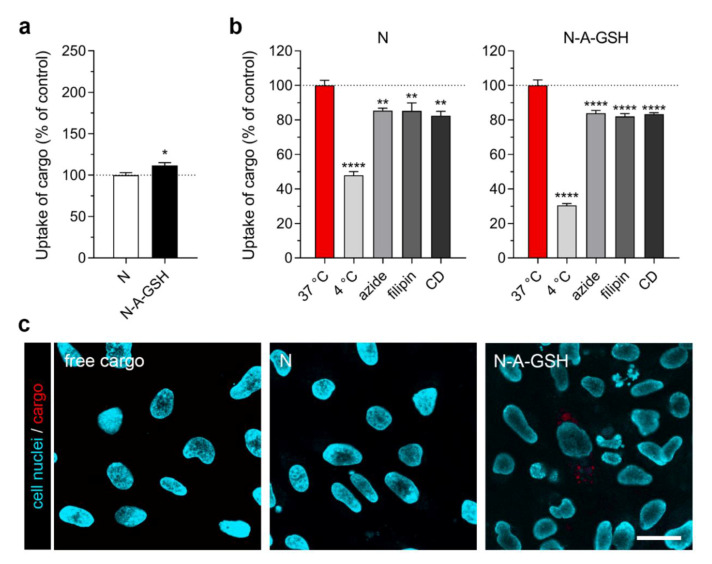
Cellular uptake of niosome cargo in hCMEC/D3 brain endothelial cells after 4 h of incubation. (**a**) Uptake of cargo loaded in non-targeted (N) and alanine-glutathione targeted (N-A-GSH) niosomes. Values presented are means ± SEM. Statistical analysis: unpaired t test; * *p* < 0.05; *n* = 6. (**b**) Effect of temperature and treatment with sodium-azide (1 mg/mL), filipin (5 µg/mL) or cytochalasin D (CD; 0.125 µg/mL) on the cellular uptake of cargo. Values presented are means ± SEM. Statistical analysis: one-way ANOVA followed by Dunnett’s posttest; ** *p* < 0.01, **** *p* < 0.0001 compared to the first column of each niosome group; *n* = 6. (**c**) Live cell visualization of cargo taken up by hCMEC/D3 cells. Free cargo: cargo not loaded in niosomes. Bar: 25 µm.

We have previously demonstrated that N-A-GSH niosomes enhace cargo uptake in primary rat brain endothelial cells [21]. Here, the same NPs significantly increased the uptake of EBA in the widely used human brain endothelial cell line hCMEC/D3, but this increase was moderate (12% compared to non-targeted niosomes, Figure 6a). In concordance with our results on rat brain endothelial cells, reduced levels of cellular uptake could be observed when the experiment was performed at 4 °C, or hCMEC/D3 cells were treated with sodium-azide, filipin or CD (N_4°C_: 48%, N_azide_: 85%; N_filipin_: 85%, N_CD_: 82%; N-A-GSH_4°C_: 30%, N-A-GSH_azide_: 84%, N-A-GSH_filipin_: 82%, N-A-GSH_CD_: 83%; Figure 5b). These data suggest an active process and a partial role of endocytosis in the uptake of cargo in hCMEC/D3 cells, too.

We also visualized the uptake of mCherry cargo in hCMEC/D3 cells, shown in Figure 6c. Overall, a limited amount of cargo could be observed inside cells by confocal microscopy. Red fluorescence was not detectable in cells treated with either free cargo or non-targeted niosomes. The number of visible particles was higher in hCMEC/D3 cells treated with alanine-glutathione dual-targeted niosomes, but interestingly, this was limited to few cells (Figure 6c).

### 3.7. Cellular Uptake of Cargo: Differentiated SH-SY5Y Neuronal Cells

**Figure 7:**
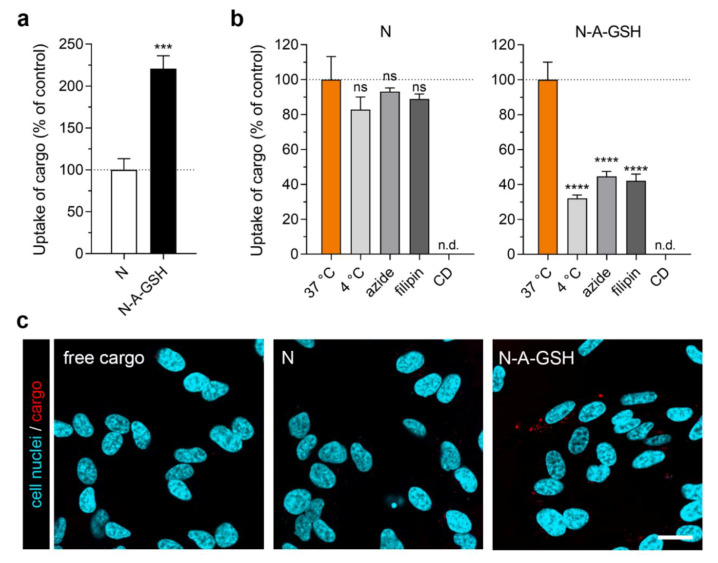
Cellular uptake of niosome cargo in differentiated SH-SY5Y neuronal cells after 4 h of incubation. (**a**) Uptake of cargo loaded in non-targeted (N) and alanine-glutathione targeted (N-A-GSH) niosomes. Values presented are means ± SEM. Statistical analysis: unpaired *t* test; *** *p* < 0.001; *n* = 6. (**b**) Effect of temperature and treatment with sodium-azide (1 mg/mL) or filipin (5 µg/mL) on the cellular uptake of cargo. The effect of cytochalasin D (CD) could not be determined (n.d.; see text). Values presented are means ± SEM. Statistical analysis: one-way ANOVA followed by Dunnett’s posttest; **** *p* < 0.0001, ns: not significant (*p* > 0.05) compared to the first column of each niosome group; *n* = 6. (**c**) Live cell visualization of cargo taken up by SH-SY5Y cells. Free cargo: cargo not loaded in niosomes. Bar: 25 µm.

We observed the biggest difference in the uptake of cargo between N-A-GSH and N groups in differentiated SH-SY5Y human neuronal cells (N-A-GSH: 221% compared to N; Figure 7a). In the case of cells treated with N-A-GSH, the uptake of cargo was greatly decreased at 4 °C and by sodium-azide or filipin (N-A-GSH_4°C_: 32%, N-A-GSH_azide_: 45%; N-A-GSH_filipin_: 42%; Figure 7b). In the case of cells treated with non-targeted niosomes, however, none of the treatments reduced the uptake statistically significantly (N_4°C_: 83%, N_azide_: 93%; N_filipin_: 89%; *p* > 0.05). Due to high toxicity in repeated experiments, we could not determine the effect of cytochalasin D on the uptake process in SH-SY5Y cells.

Live cell visualization of mCherry cargo taken up by SH-SY5Y cells is shown in Figure 7c. Red fluorescence was again barely detectable in cells treated with either free cargo or non-targeted niosomes. In accordance with the spectrophotometry data in Figure 7a, the highest amount of mCherry cargo could be seen in cells treated with alanine-glutathione dual-targeted niosomes.

### 3.8. Permeability of Cargo Across the Blood–Brain Barrier (BBB) Co-Culture Model

**Figure 8:**
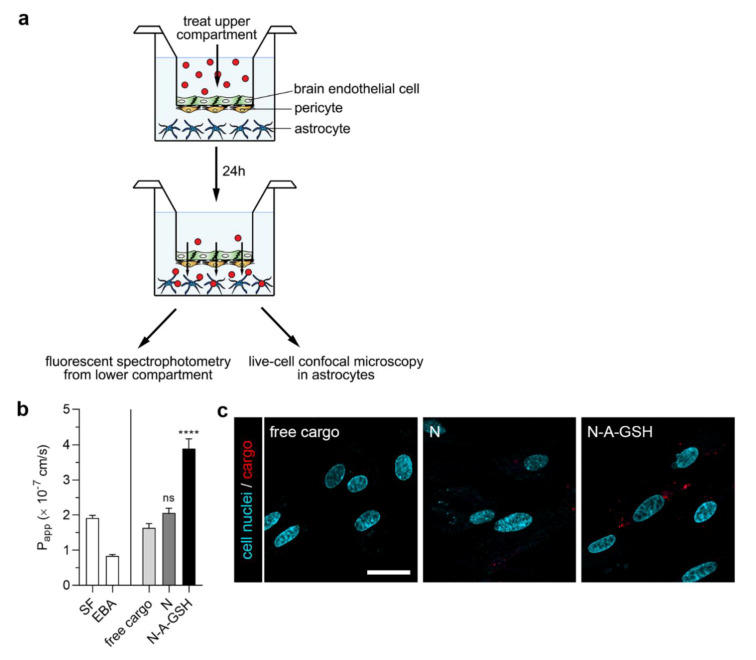
(**a**) Schematic drawing of the BBB co-culture model and the workflow of the experiment. (**b**) 24-h permeability of mCherry cargo across the BBB model. P_app_: apparent permeability coefficient; SF: sodium fluorescein; EBA: Evans blue-albumin complex; free cargo: mCherry not loaded in niosomes; N: non-targeted niosome; N-A-GSH: alanine-glutathione dual-targeted niosome. Values presented are means ± SEM. Statistical analysis: one-way ANOVA followed by Dunnett’s posttest; **** *p* < 0.0001, ns: not significant (*p* > 0.05) compared to the free cargo group; *n* = 3–8. (**c**) Live cell visualization of mCherry cargo taken up by living astrocytes after having crossed the BBB model. Bar: 25 µm.

Figure 8a shows a schematic drawing of the BBB co-culture model and the experimental setup we used to assess the penetration of cargo across the BBB and its subsequent uptake by astrocytes. To evaluate the integrity of the model, we also measured the penetration of the small paracellular permeability marker sodium fluorescein (SF, 376 Da) and the large marker EBA (67 kDa) across the BBB. As determined in the 24-h experiment, P_app_ values were 1.92 × 10^−7^ cm/s for SF and 8.39 × 10^−8^ cm/s for EBA (Figure 8b, left panel). These values reflect a tight barrier and are in agreement with our previous results [15,21]. To further substantiate that the differences between cargo P_app_ values are not due to loss of tight junction integrity, we provide TEER values measured before and after the experiment in Supplementary Table S2.

The permeability of mCherry cargo encapsulated in non-targeted niosomes (2.06 × 10^−7^ cm/s) was higher, although statistically not significantly, compared to non-encapsulated (free) mCherry transfer (1.63 × 10^−7^ cm/s; Figure 8b, right panel). Labeling niosomes with the combinaton of alanine and glutathione further enhanced the penetration of cargo across the BBB model (3.89 × 10^−7^ cm/s), which is a 2.39-fold and 1.89-fold increase compared to the free cargo and N groups, respectively.

We also visualized mCherry cargo that had crossed the BBB model inside living astrocytes by confocal microscopy (Figure 8c). The red fluorescent signal of mCherry could be observed in astroglial cells at the end of the 24-h permeability experiment with N-A-GSH niosomes, indicating not only the penetration of cargo across the BBB, but also its uptake by astrocytes. Considerably less mCherry signal could be seen in the non-targeted niosome group, while in the case of free mCherry, the red fluorescent signal was barely detectable in astrocytes.

## 4. Discussion

The delivery of drugs, especially biopharmacons, across the BBB and into the cells of the NVU is still a limiting step in the treatment of neurological diseases. Here, we are the first to present a targeted NP-based approach that enabled the delivery of the 67 kDa EBA and the 26.7 kDa mCherry cargo proteins not only into brain endothelial cells, but also into pericytes, astrocytes and differentiated neuronal cells.

### 4.1. Alanine-Glutathione Dual-Labeling of NPs as a Strategy for Entry in the Cells of the Neurovascular Unit

In our previous study [21], we identified the amino acid alanine as a potent BBB targeting molecule of NPs. We have also shown that niosomes labeled with the right combination of SLC ligands greatly and specifically enhance the penetration of cargo across the BBB in vitro and in vivo [21]. According to our hypothesis, carriers of alanine and glutathione are promising targets of NPs in other cells of the NVU too.

Alanine enters the brain via neutral amino acid transporters ASCT1, ASCT2, SNAT1, SNAT2 and SNAT5 [9]. Here, we verified that all five genes encoding these SLCs are also expressed in cultured pericytes, astrocytes and neurons, which is in agreement with larger-scale RNA-Seq data [46,47,48,49] and functional analyses [50] from the literature. In concordance with qRT-PCR data on rodent and human brain endothelial cells [51,52], including our own results ([15]; also shown in Figure 1b), we found *SNAT2* (*SLC38A2*) to also be highly expressed in pericytes and astrocytes. Regarding glutathione, there is evindence for a luminal, Na^+^-dependent, carrier-mediated transport of this tripeptide in brain endothelial cells and astrocytes [53,54,55]. The exact mechanism, including the transporter(s) by which it enters the brain and the cells of the NVU, however, remains to be elucidated.

### 4.2. Alanine-Glutathione Targeted Niosomes Enchance the Delivery of a Large Protein Cargo into the Cells of the Neurovascular Unit

In all NVU cell types tested, significantly more cargo was taken up by cells when it was encapsulated in alanine-glutathione dual-targeted niosomes compared to non-targeted NPs. This increase was more than 2-fold in pericytes (Figure 4a) and cargo was well visualized in both N and N-A-GSH groups (Figure 4c). In addition to endocytosis or fusion of NPs with the plasma membrane, this process may be also related to the fact that pericytes are capable of phagocytosis [56,57]. Although these cells regulate many important CNS-specific processes in health and disease [58,59,60,61], we found no other reports on targeted drug delivery to brain pericytes, only to pericytes in the periphery. A study, for example, describes a TH10 peptide-targeted, docetaxel-loaded polymeric NP that triggered pericyte apoptosis in a lung metastasis of melanoma in mice, resulting in an anti-angiogenic effect in the tumor and longer survival time [62].

Astroglial cells and their tumors, on the other hand, are widely recognised as drug targets. Labeling polymeric NPs with glutathione, for example, increased the delivery of chemotherapeutic drugs paclitaxel and docetaxel to C6 and RG2 rat glioma cells [63,64]. In our study, primary rat astrocytes also showed a preference for the uptake of N-A-GSH niosomes (128% compared to non-targeted niosomes, Figure 5a), but internalised cargo in both NP groups as observed by confocal microscopy (Figure 5c). Our finding, that the cargo of non-targeted niosomes was visible in astrocytes in contrast to brain endothelial cells or neurons, might be related to the observation that cultured astrocytes can be efficiently lipofected [65,66]. Another important glial cell type, microglial cells, well-known for their ability to internalize nanosized particles and exosomes by phagocytosis or macropinocytosis, were not investigated in our study. In this cell type SLC transporters, which may mediate targeted nanoparticle uptake, are less known and remain to be investigated.

Glutathione PEGylation of liposomes, an approach pioneered by the group of Pieter Gaillard, enhanced the delivery of doxorubicin, ribavirin and methylprednisolone into hCMEC/D3 human brain endothelial cells and across the BBB in preclinical studies [18,19]. Furthermore, our group has also demonstrated the ability of solid NPs labeled with biotinylated glutathione to enter hCMEC/D3 cells [35]. In our experiments, N-A-GSH niosomes also significantly increased cargo delivery into hCMEC/D3 cells (Figure 6a), but this effect was modest compared to our previous results with primary RBECs [21]. A recent article highlighting major differences in the composition of the endo-lysosomal system between hCMEC/D3 and primary porcine brain endothelial cells might support our observation that NPs interact differently with hCMEC/D3 cells and primary RBECs [67].

Interestingly, we measured the greatest difference between the uptake of N-A-GSH and non-targeted niosomes in differentiated human SH-SY5Y neuronal cells (Figure 7a). This 2.2-fold increase in cargo delivery is in agreement with a recent study in which GSH-functionalized polymeric NPs were able to deliver curcumin with comparable efficacy into SK-N-SH neuronal cells, a maternal cell line of SH-SY5Y [68]. Moreover, an increase in the uptake of fluorescein-loaded albumin NPs in a mixed neuron-glia co-culture were described when the particles were labeled with glutathione [69]. We also observed an increase in cargo delivery by our dual-labeled NPs, while cargo fluorescence was barely detectable in SH-SY5Y cells treated with either free cargo or non-targeted niosomes (Figure 7c).

### 4.3. The Uptake of Targeted Niosomes is Energy-Dependent and is Partially Mediated by Endocytosis

Low temperature and sodium azide are widely used to investigate the energy-dependency of NP internalization. These treatments inhibit all active uptake processes and mitochondrial ATP production in cells, respectively [44]. Here, the uptake of N-A-GSH was decreased at 4 °C or in response to sodium azide in each cell type tested (Figure 4b, Figure 5b, Figure 6b and Figure 7b).

To determine whether endocytosis plays a role in the mechanism of cellular uptake, we used two inhibitors, filipin and cytochalasin D. Filipin interacts with cholesterol in biological membranes and blocks lipid raft/caveolae-mediated endocytosis, while cytochalasin D is an actin polymerization-blocking agent, thereby inhibiting all major endocytic routes [45]. Pre-treatment of cells with either filipin or cytochalasin D significantly lowered the uptake of N-A-GSH in all cell types tested (Figure 4b, Figure 5b, Figure 6b and Figure 7b). These data are consistent with our previous results on primary RBECs [21] as well as reports on GSH-targeted particles in hCMEC/D3 [70] and SH-SY5Y cell lines [69]. Therefore, we conclude that the uptake process of N-A-GSH niosomes is active and is partially mediated by endocytosis in each NVU cell type tested. The cellular uptake of niosomes is likely to be mediated by other, non-endocytic mechanisms parallelly, such as fusion of the nanovesicles with the plasma membrane of cells as we previously showed in primary brain endothelial cells [21]. However, this phenomenon remains to be further studied in other NVU cells.

### 4.4. Dual-Labeling of Niosomes with Alanine and Glutathione Enchances Cargo Delivery Across the BBB and Subsequently to Astrocytes

Despite the well-known fact that multiple cell types surround brain capillaries, very few studies actually focus on how NPs interact with these cells once they have crossed the BBB. Gromnicova et al., for example, reported the passage of 4-nm gold NPs labeled with the SLC ligand glucose across hCMEC/D3 cells and into primary human astrocytes in a 3D collagen gel in vitro model [24]. Furthermore, they also demonstrated the presence of these gold NPs in brain microvessel pericytes, glial cells and neurons after intravenous injections in rats [71]. Others showed the entry of transferrin-penetratin dual-labeled liposomes into neurons after translocating the BBB using an in vitro model and in mice [72].

In our work, we tested whether N-A-GSH niosomes are able to deliver mCherry cargo into astroglial cells after having crossed the BBB in vitro. For this purpose, we used our well-characterized BBB co-culture model consisting of primary RBECs, pericytes and astrocytes [15,27]. The static environment is a limitation of culture insert-based methods, although we performed the uptake and permeability experiments on a horizontal shaker to limit the unstirred water layer effect. The transfer of permeability marker molecules was very low in the 24-h experiment, reflecting good barrier properties. Encapsulation of cargo into N-A-GSH niosomes elevated its penetration across the BBB by about 2-fold compared to free cargo and N groups (Figure 8b), which is in agreement with our previous results [21]. After 24 h, we were able to visualize the fluorescent protein cargo inside living astroglial cells in the N-A-GSH group (Figure 8c). This indicates that niosomes dual-targeted with alanine and glutathione carrying mCherry cargo crossed the BBB and were subsequently taken up by astrocytes. Interestingly, a more substantial difference in cargo uptake between N-A-GSH and N groups could be observed in astrocytes after the permeability experiment (Figure 8c) compared to the astrocytic uptake experiment in Figure 5c. An explanation to this might be that brain endothelial cells control primarily the amount of material that is available for astrocytic uptake at the bottom compartment (Figure 8b). Therefore, we believe that the difference we see between groups in Figure 8c could be the combined effect of targeted niosomes interacting first with brain endothelial and then with astrocytes.

## 5. Conclusions

In this in vitro study, we focused on the interaction of targeted vesicular nanoparticles with multiple cell types of the neurovascular unit. We showed that decorating the surface of niosomes with the combination of alanine and glutathione enhanced cargo delivery into cultured brain endothelial cells, pericytes, astrocytes and differentiated neuronal cells compared to non-targeted niosomes. Alanine-glutathione dual-labeling of nanoparticles was most effective in primary rat pericytes and SH-SY5Y neuronal cells, increasing the cellular uptake of EBA, a 67 kDa protein cargo, by more than 2-fold. Moreover, we revealed that the cellular uptake of niosomes is energy-dependent and is partially mediated by endocytosis. Finally, we demonstated the ability of our dual-targeted nanovesicles to deliver the 26.7 kDa protein cargo mCherry into astroglial cells after crossing layers of brain endothelial cells and pericytes in a co-culture model of the blood–brain barrier. Our data suggest that alanine-glutathione dual-labeling of nanoparticles can potentially be exploited to deliver drugs, even biopharmacons, across the blood–brain barrier and into other therapeutically relevant cell types in the brain.

## Supplementary Materials

The following are available online at https://www.mdpi.com/1999-4923/12/7/635/s1, Figure S1: Size distribution profile of serum (10% FBS in PBS) and non-targeted (N) as well as alanine-glutathione dual-targeted (N-A-GSH) niosomes in serum. Graphs and summaries were made using the Malvern Zetasizer Nano ZS software. Table S1: Values of cellular uptake experiments shown in Figure 4a, Figure 5a, Figure 6a and Figure 7a: EBA cargo taken up by primary rat pericytes (RPC), primary rat astrocytes (RAC), hCMEC/D3 endothelial cells and SH-SY5Y neurons normalized to EBA cargo encapsulated in non-targeted (N) and alanine-glutathione dual-targeted (N-A-GSH) niosomes in the 10 mg/mL treatment solution (left panel) and the mean number of cells/well ± SD (right panel). Figure S2: Fluorescent, brightfield and merged channels of confocal microscopy images presented in a) Figure 4c, b) Figure 5c, c) Figure 6c, d) Figure 7c,e) Figure 8c. Scale bar: 25 μm. Table S2: Transendothelial electrical resistance (TEER) values (Ω × cm2) in the BBB co-culture model before and after the 24-h permeability experiment shown in Figure 8. N: non-targeted niosome group; N-A-GSH: alanine-glutathione dual-targeted niosome group.
