# Supplementary material for: Combination of Alanine and Glutathione as Targeting Ligands of Nanoparticles Enhances Cargo Delivery into the Cells of the Neurovascular Unit
Source: Pharmaceutics. 2020 Jul 7;12(7):635. doi: 10.3390/pharmaceutics12070635 (PMC7407318; doi:10.3390/pharmaceutics12070635)
Supplement: Supplementary file 1 [file pharmaceutics-12-00635-s001.pdf]

pharmaceutics

Supplementary Materials: Combination of Alanine and Glutathione as Targeting Ligands of Nanoparticles Enhances Cargo Delivery into the Cells of the Neurovascular Unit

Gergő Porkoláb, Mária Mészáros, András Tóth, Anikó Szecskó, András Harazin, Zsolt Szegletes, Györgyi Ferenc, András Blastyák, Lajos Mátés, Gábor Rákhely, Mária A. Deli and Szilvia Veszelka

Contents:

Page 2

Supplementary Figure S1. Size distribution profile of serum (10% FBS in PBS) and non-targeted (N) as well as alanine-glutathione dual-targeted (N-A-GSH) niosomes in serum. Graphs and summaries were made using the Malvern Zetasizer Nano ZS software.

Page 3

Supplementary Table S1. Values of cellular uptake experiments shown in Figures 4a, 5a, 6a and 7a: EBA cargo taken up by primary rat pericytes (RPC), primary rat astrocytes (RAC), hCMEC/D3 endothelial cells and SH-SY5Y neurons normalized to EBA cargo encapsulated in non-targeted (N) and alanine-glutathione dual-targeted (N-A-GSH) niosomes in the 10 mg/mL treatment solution (left panel) and the mean number of cells / well ± SD (right panel).

Pages 4-8 (continued)

Supplementary Figure S2. Fluorescent, brightfield and merged channels of confocal microscopy images presented in **a)** Figure 4c, **b)** Figure 5c, **c)** Figure 6c, **d)** Figure 7c and **e)** Figure 8c. Scale bar: 25 μm.

Page 9

Supplementary Table S2. Transendothelial electrical resistance (TEER) values ($\Omega \times cm^2$) in the BBB co-culture model before and after the 24-hour permeability experiment shown in Figure 8. N: non-targeted niosome group; N-A-GSH: alanine-glutathione dual-targeted niosome group.

Serum (10% FBS in PBS)

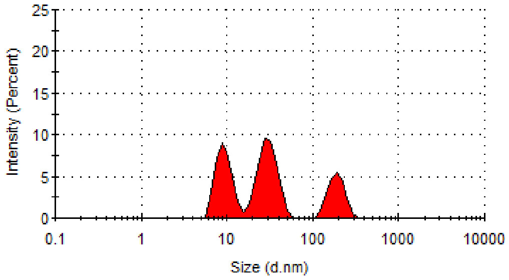

	Size (d.nm):	% Intensity:	St Dev (d.nm):
Peak 1:	30.27	43.8	7.510
Peak 2:	9.391	34.9	2.033
Peak 3:	191.3	21.3	39.99

N 2 mg/mL in serum

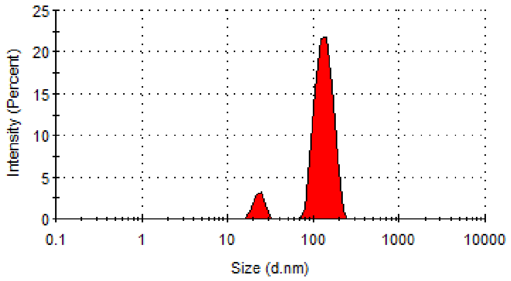

	Size (d.nm):	% Intensity:	St Dev (d.nm):
Peak 1:	135.0	91.4	29.30
Peak 2:	23.49	8.6	3.200

N 10 mg/mL in serum

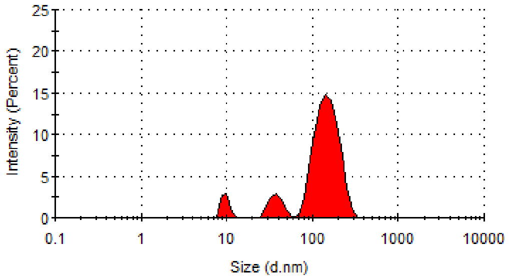

	Size (d.nm):	% Intensity:	St Dev (d.nm):
Peak 1:	152.6	84.2	46.58
Peak 2:	38.13	9.5	6.354
Peak 3:	9.663	6.3	0.9489

N-A-GSH 2 mg/mL in serum

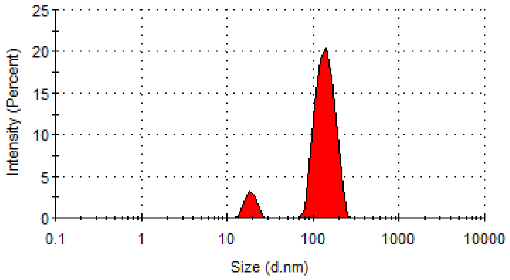

	Size (d.nm):	% Intensity:	St Dev (d.nm):
Peak 1:	141.1	91.7	33.41
Peak 2:	18.92	8.3	2.613

N-A-GSH 10 mg/mL in serum

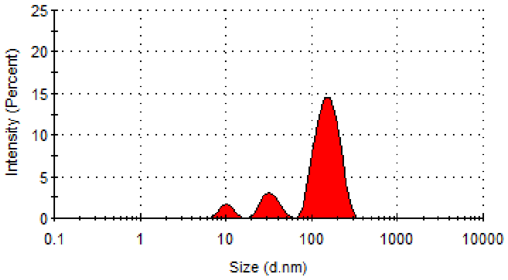

	Size (d.nm):	% Intensity:	St Dev (d.nm):
Peak 1:	158.1	81.7	47.04
Peak 2:	33.34	12.6	7.336
Peak 3:	10.27	5.6	1.720

Supplementary Figure S1. Size distribution profile of serum (10% FBS in PBS) and non-targeted (N) as well as alanine-glutathione dual-targeted (N-A-GSH) nanoparticles in serum. Graphs and summaries were made using the Malvern Zetasizer Nano ZS software.

pharmaceutics

MDPI

Supplementary Table S1. Values of cellular uptake experiments shown in Figures 4a, 5a, 6a and 7a: EBA cargo taken up by primary rat brain pericytes (RPC), primary rat astrocytes (RAC), hCMEC/D3 endothelial and SH-SY5Y neuronal cells normalized to EBA cargo encapsulated in non-targeted (N) and alanine-glutathione dual-targeted (N-A-GSH) niosomes in the 10 mg/mL treatment solution (left panel) and mean number of cells / well ± SD (right panel).

		mg EBA cargo in cells / mg EBA cargo in nanoparticle treatment solution		Mean number of cells per well ± SD
Treatment group	Replicates	N	N-A-GSH	
RPC	1	0.002619	0.007899	53 125 ± 10 017
	2	0.002724	0.006017	
	3	0.004046	0.005665	
	4	0.003509	0.007196	
	Mean	0.003224	0.006694	
	SD	0.000676	0.001036	
RAC	1	0.001350	0.001685	147 321 ± 26 361
	2	0.001128	0.001518	
	3	0.001037	0.001437	
	4	0.001173	0.001474	
	5	0.001177	0.001651	
	6	0.001415	-	
	Mean	0.001213	0.001553	
	SD	0.000142	0.000109	
hCMEC/D3	1	0.002971	0.003050	126 176 ± 17 759
	2	0.002793	0.003014	
	3	0.002607	0.002808	
	4	0.002528	0.002854	
	5	0.002499	0.002970	
	6	0.002883	0.003470	
	Mean	0.002713	0.003028	
	SD	0.000196	0.000236	
SH-SY5Y	1	0.014714	0.033570	341 967 ± 55 533
	2	0.011129	0.038071	
	3	0.013619	0.044125	
	4	0.021349	0.036816	
	5	0.023089	0.049380	
	6	0.025938	-	
	Mean	0.018306	0.040392	
	SD	0.005946	0.006313	

pharmaceutics

MDPI

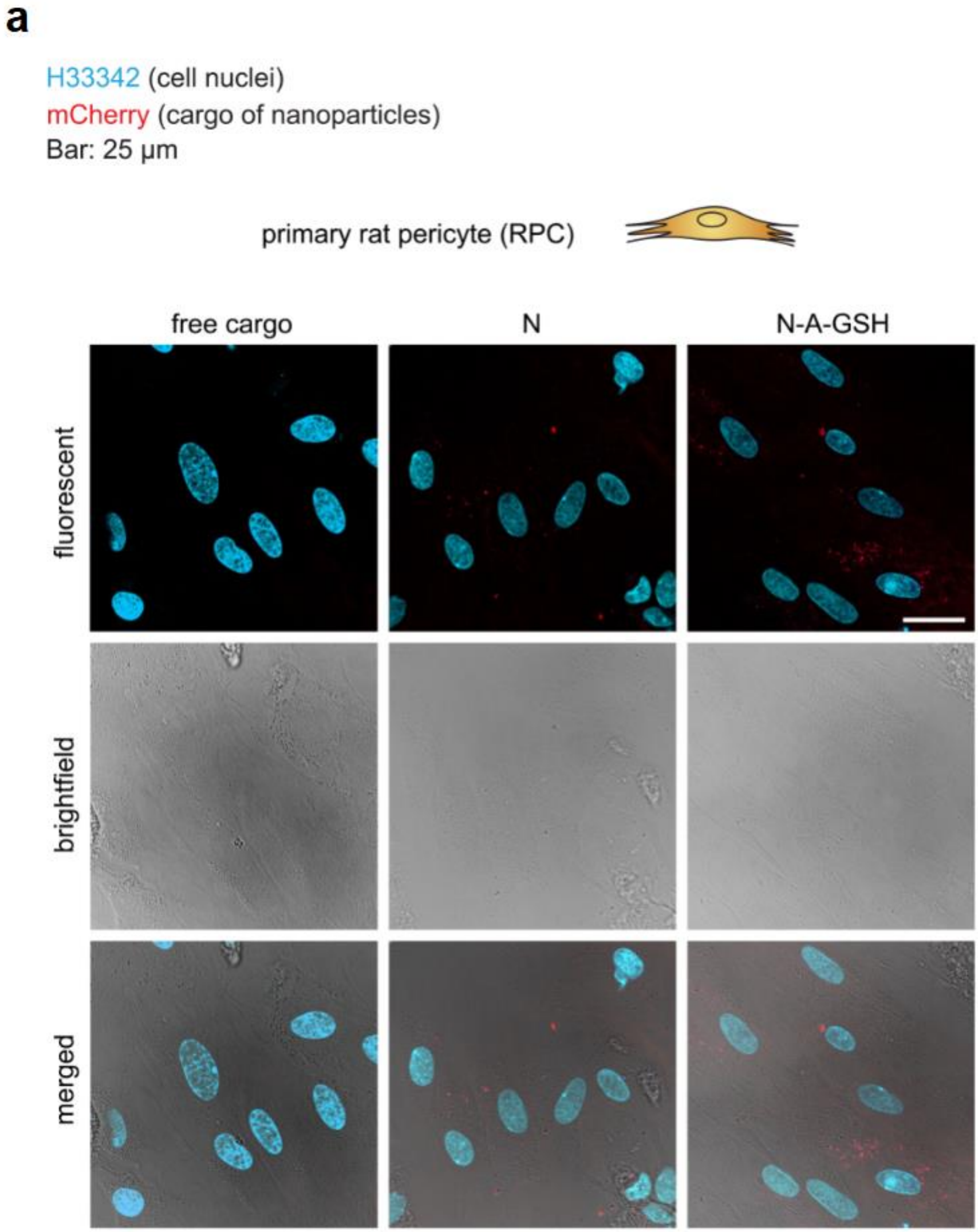

Supplementary Figure S2. Fluorescent, brightfield and merged channels of confocal microscopy images presented in **a)** Figure 4c, **b)** Figure 5c, **c)** Figure 6c, **d)** Figure 7c and **e)** Figure 8c. Scale bar: 25 µm. (continued on pages 4-8)

pharmaceutics

MDPI

Supplementary Figure S2. (continued)

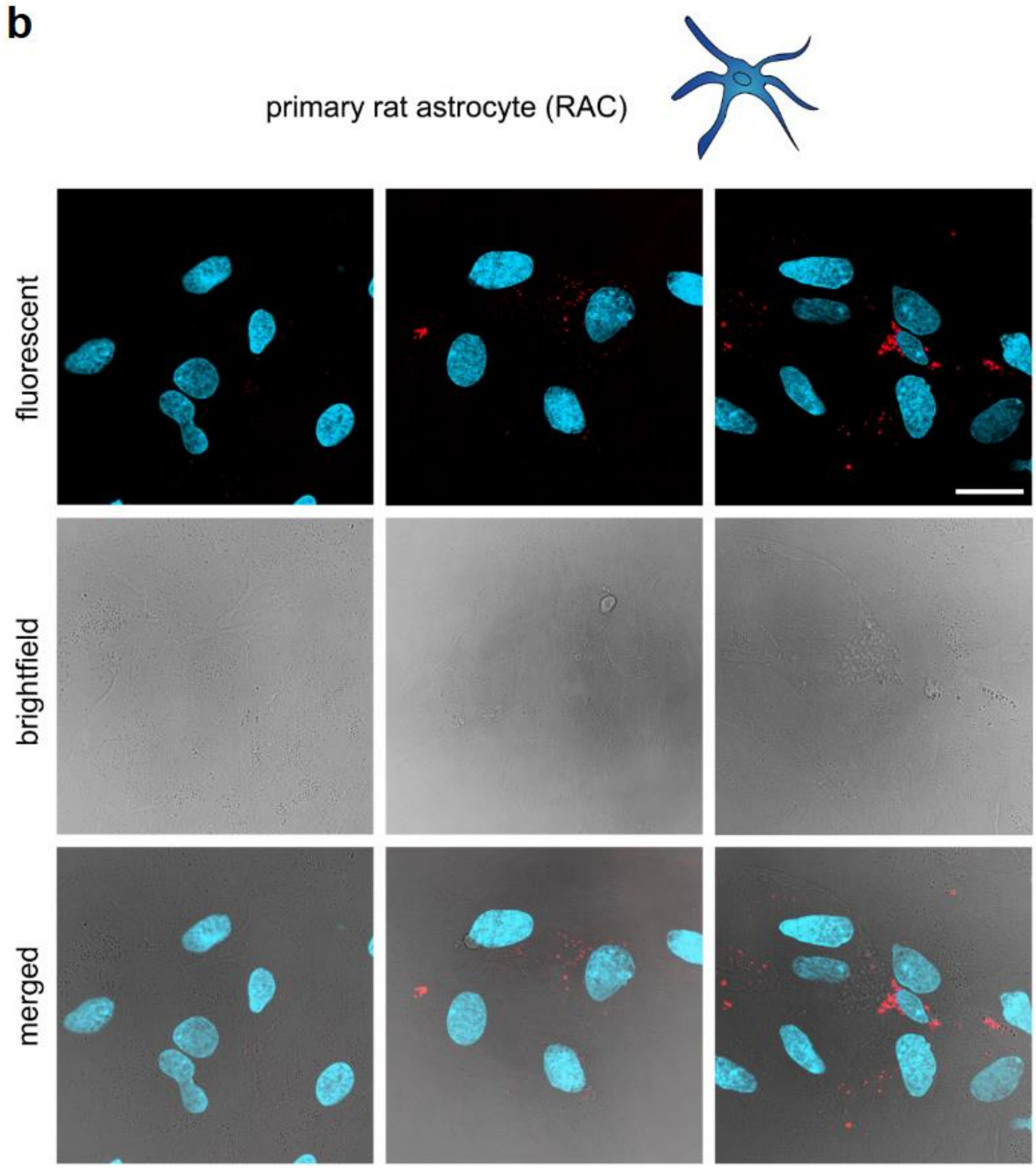

pharmaceutics

MDPI

Supplementary Figure S2. (continued)

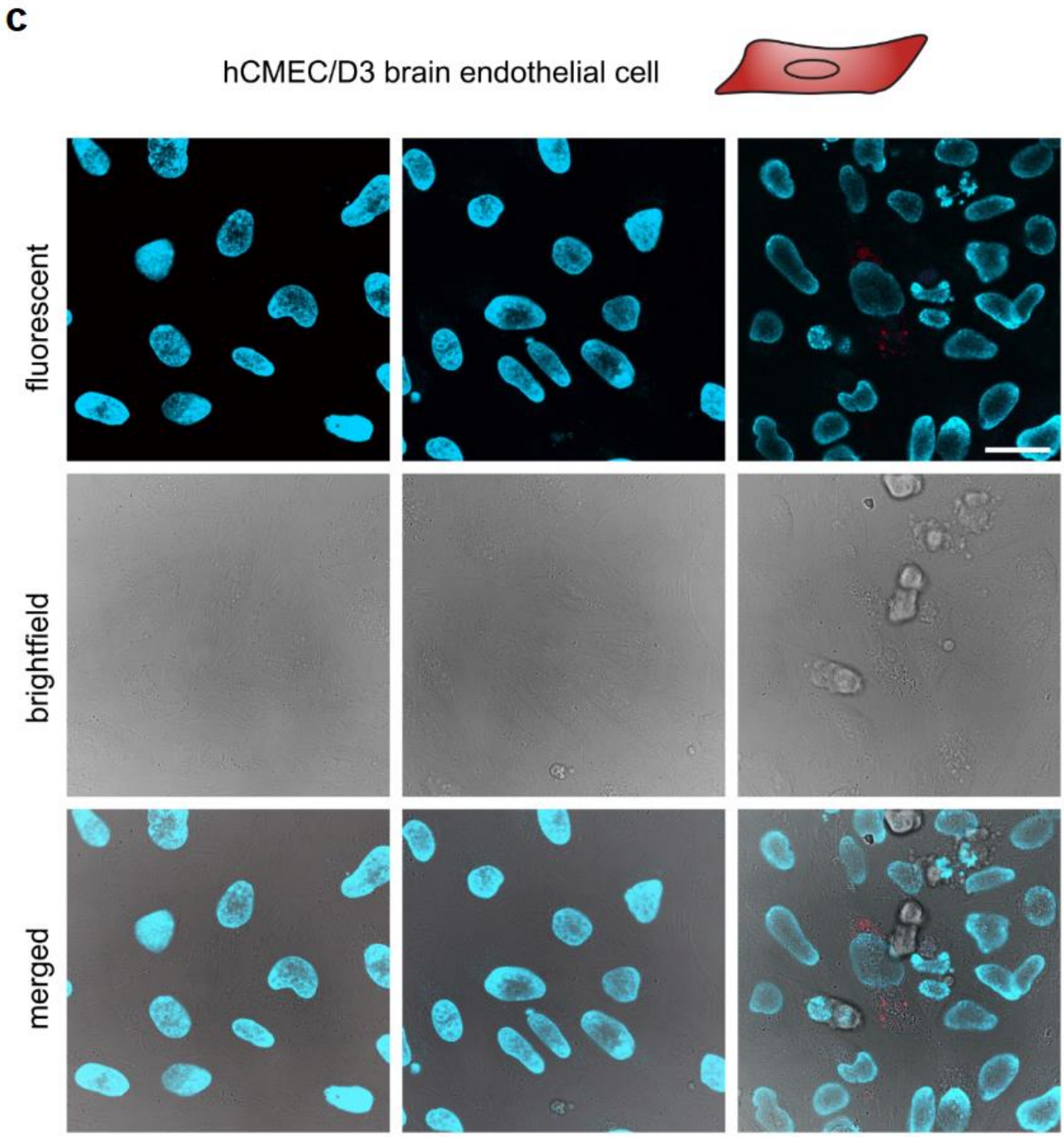

pharmaceutics

MDPI

Supplementary Figure S2. (continued)

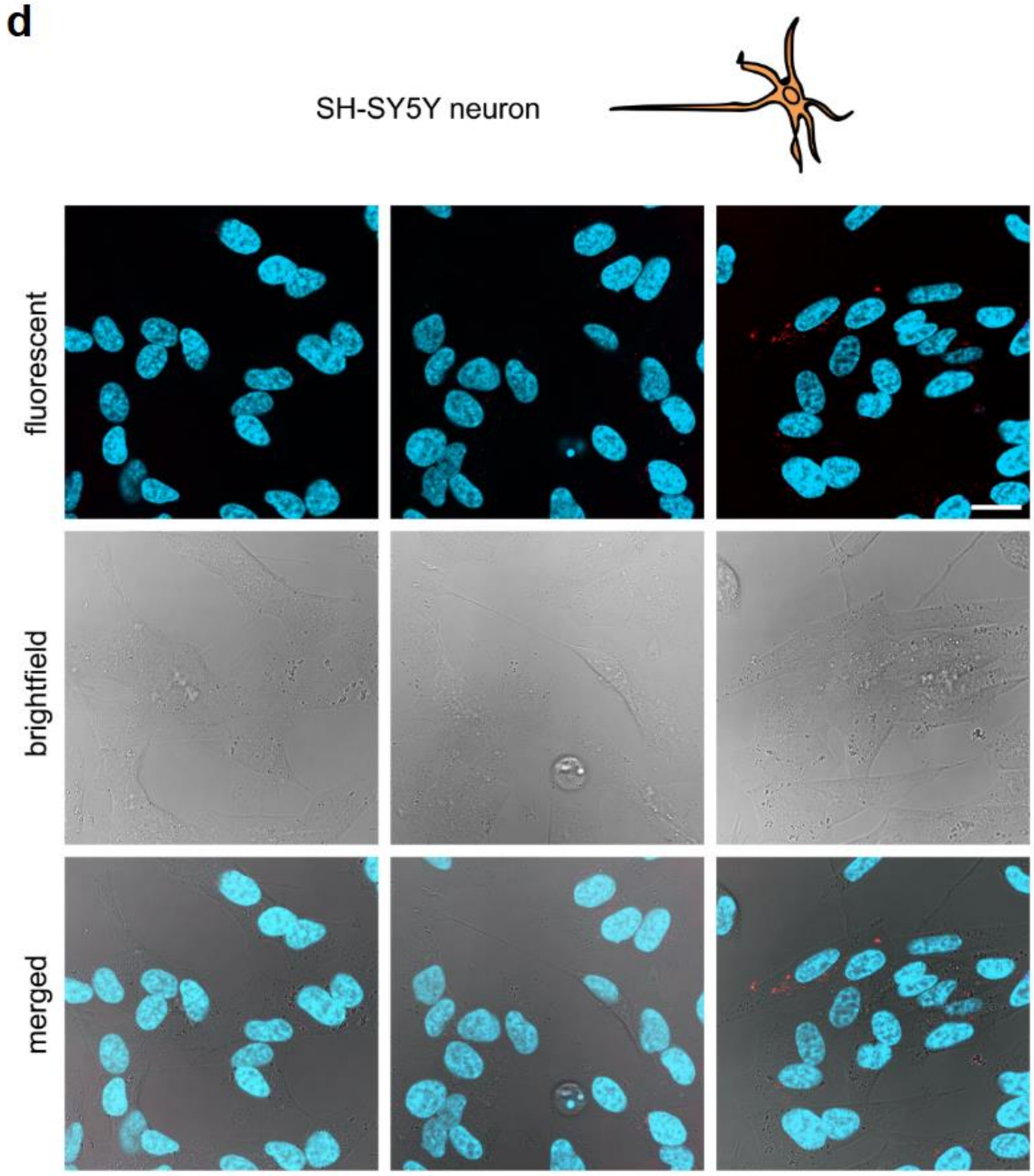

pharmaceutics
MDPI

Supplementary Figure S2. (last panel)

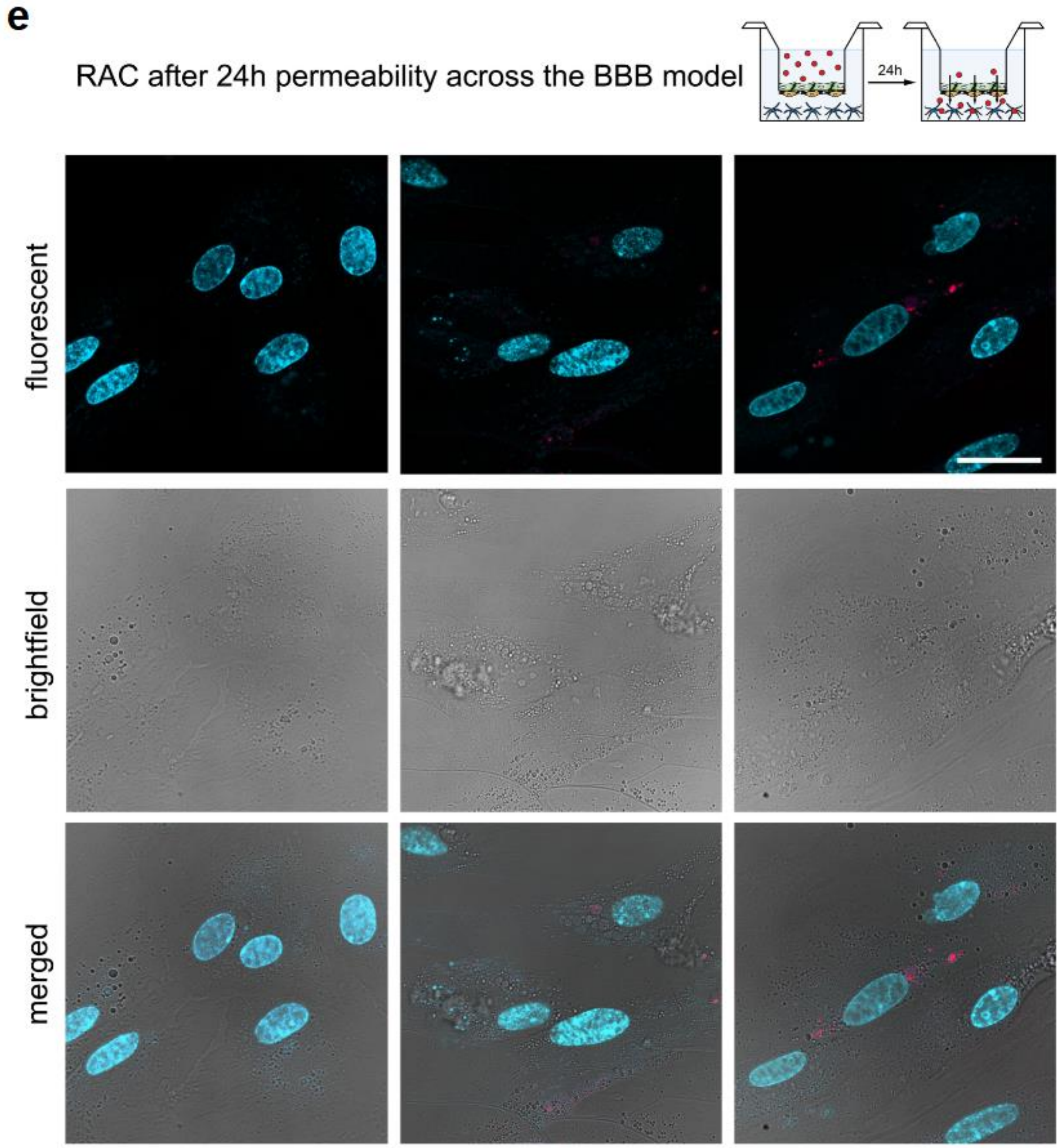

The authors would like to note that the quality of images is affected when compressed into a PDF document. Full-size images can be provided by the corresponding authors upon request.

pharmaceutics

MDPI

Supplementary Table S2. Transendothelial electrical resistance (TEER) values (Ω × cm^2) in the BBB co-culture model before and after the 24-hour permeability experiment shown in Figure 8. N: non-targeted niosome group; N-A-GSH: alanine-glutathione dual-targeted niosome group.

Treatment group	Replicates	Before	After	Difference
N	1	302	226	76
	2	295	240	55
	3	286	210	76
	4	286	194	92
	Mean	292.3	217.5	74.8
	SD	7.8	19.9	15.2
N-A-GSH	1	318	251	67
	2	306	234	72
	3	300	218	82
	4	279	220	59
	Mean	300.8	230.8	70.0
	SD	16.3	15.3	9.6
free mCherry	1	286	221	65
	2	266	212	54
	3	295	209	86
	Mean	282.3	214.0	68.3
	SD	14.8	6.2	16.3
